# Audiotactile interaction can change over time in cochlear implant users

**DOI:** 10.3389/fnhum.2014.00316

**Published:** 2014-05-22

**Authors:** Simon P. Landry, Jean-Paul Guillemot, François Champoux

**Affiliations:** ^1^Centre de Recherche en Neuropsychologie Expérimentale et Cognition, Université de MontréalMontréal, QC, Canada; ^2^Département de Kinanthropologie, Université du Québec à MontréalMontréal, QC, Canada; ^3^Institut Raymond-Dewar, Centre de Recherche Interdisciplinaire en Réadaptation du Montréal MétropolitainMontréal, QC, Canada; ^4^École d’Orthophonie et d’Audiologie, Faculté de Médecine, Université de MontréalMontréal, QC, Canada

**Keywords:** audiotactile interaction, multisensory interactions, cochlear implant, parchment-skin illusion, sensory deprivation, cross-modal plasticity, deafness, hearing loss

## Abstract

Recent results suggest that audiotactile interactions are disturbed in cochlear implant (CI) users. However, further exploration regarding the factors responsible for such abnormal sensory processing is still required. Considering the temporal nature of a previously used multisensory task, it remains unclear whether any aberrant results were caused by the specificity of the interaction studied or rather if it reflects an overall abnormal interaction. Moreover, although duration of experience with a CI has often been linked with the recovery of auditory functions, its impact on multisensory performance remains uncertain. In the present study, we used the parchment-skin illusion, a robust illustration of sound-biased perception of touch based on changes in auditory frequencies, to investigate the specificities of audiotactile interactions in CI users. Whereas individuals with relatively little experience with the CI performed similarly to the control group, experienced CI users showed a significantly greater illusory percept. The overall results suggest that despite being able to ignore auditory distractors in a temporal audiotactile task, CI users develop to become greatly influenced by auditory input in a spectral audiotactile task. When considered with the existing body of research, these results confirm that normal sensory interaction processing can be compromised in CI users.

## INTRODUCTION

Audiovisual interactions have been extensively studied in the hearing. Resulting evidence put forth that interaction between senses enhances overall perceptual accuracy and saliency through cooperative advantages in congruent situations (e.g., Calvert and Thesen, 2004; Stein and Stanford, 2008) and provides the redundancy of cues that is necessary to fully characterize objects in our environment (e.g., Driver and Noesselt, 2008). Audiovisual processing has also been widely examined in cochlear implant (CI) users (e.g., Tyler et al., 1997; Kaiser et al., 2003; Geers, 2004; Moody-Antonio et al., 2005; Champoux et al., 2009; Tremblay et al., 2010; Landry et al., 2012). However, multisensory interaction in CI users outside of the audiovisual domain has not received the same attention. This neglect is unfortunate as it has been recently proposed that several unexplained day-to-day life difficulties observed in the deaf could be related to deficits in audiotactile processing (Nasir and Ostry, 2008).

The sense of touch can be altered if another sense is simultaneously stimulated. Motivated by the fact that tactile and auditory modalities are both sensitive to environmental oscillations, interactions between these modalities have recently gained attention from some researchers (Yau et al., 2009a, b; Yau et al., 2010). Such multisensory interactions can be examined using different tasks. Arguably, the most robust cases of cross-modal fusion between auditory and tactile modalities are the audiotactile illusory flash effect (Hötting and Röder, 2004) and the parchment-skin illusion (Jousmäki and Hari, 1998), for the temporal domain and the spectral domain respectively. The audiotactile illusory flash effect is a non-speech illusory percept in which the simultaneous presentation of a single somatosensory stimulus with two successive sounds can lead to the perception of two distinct tactile sensations in normally hearing individuals. The parchment-skin illusion (Jousmäki and Hari, 1998) is also a non-speech illusory percept in which an amplification or reduction of high-frequency content from the sound generated by rubbing hands together results in an alteration of the experienced palmar dryness/moistness. This sound-induced alteration of touch perception appears to be a robust case of cross-modal fusion in the spectral domain (see also Guest et al., 2002; Champoux et al., 2011). The parchment-skin illusion is one of the earliest demonstrations of the importance of spectral auditory inputs on tactile perception. This task demonstrates the potential perceptual effect of auditory frequency manipulation on palmar sensation of roughness and moistness.

Recently, we investigated whether temporal audiotactile processes were disturbed in CI users (Landry et al., 2013). The audiotactile illusory flash effect was administered to a group of normally hearing individuals and a group of CI users. Control conditions revealed that auditory and tactile discrimination capabilities were identical for both groups. Whereas normally hearing individuals integrated auditory and tactile information in the context of an audiotactile illusion, CI users were not influence by the presence of auditory stimuli and thus did not perceive the audiotactile illusion. This gives strength to the hypothesis by which CI users may have audiotactile interactions deficits (Nasir and Ostry, 2008).

However, two important questions remain before such a sweeping statement can be substantiated. First, it remains unclear whether these results can be attributed exclusively to the specificity of the interaction investigated. Until now, CI user audiotactile interaction has only been examined in a temporal task (Landry et al., 2013). Thus, it remains unclear whether the observed change is related to the specificity of the interaction investigated. In order to examine whether a period of prolonged deafness can have an impact on the development of audiotactile processing at large, the performance of CI users needs to be investigated in relation to other features of the stimuli, namely spectral characteristics. Second, audiotactile performance has not yet been examined in relation to features related to cochlear implantation such as duration of CI use. In order to examine whether temporary deafness has an impact on the development of audiotactile processes at large, the performance of CI users needs to be investigated in relation to other features of the multisensory stimuli, including spectral characteristics. Moreover, duration of experience with the implant has been found to have a strong positive effect on auditory performance in various behavioral and electrophysiological tasks (e.g., Nicholas and Geers, 2006; Pantev et al., 2006). These results suggest that longer experience with the implant might help with the restoration of sensory functions after prolonged deprivation. Long-term follow-up investigations of CI patients suggest that long-term perception performance improves over time and reaches a plateau 4–5 years post-implantation (O’Donoghue et al., 1998). Furthermore, approximately 6 years of experience with the implant is required to acquire excellent results in perception performance (e.g., Allen et al., 1998; Damen et al., 2007).

In the present study, we aim at examining spectral audiotactile interaction capabilities of CI users in relation to the duration of experience with the CI. Previous investigations have demonstrated that temporal audiotactile interaction is abnormal in CI users (Landry et al., 2013), yet it is unknown if this is applicable to other domains of audiotactile interaction such as frequency. We used the parchment-skin illusion (Jousmäki and Hari, 1998) to further the knowledge of audiotactile interaction capabilities in CI users. In addition to this illusory task, control tasks provide the means for the separation of unisensory performance from multisensory performance.

## MATERIALS AND METHODS

### PARTICIPANTS

Thirty-eight participants (19 CI users and as many normal-hearing subjects matched for handedness, sex, and age) were involved in the study. CI users (6 male; mean age: 46 years; range: 22–65 years) had lost their hearing for a period of 13–53 years (see **Table 1**). The groups were comparable in regards to their educational background and occupational status. All CI users suffered from profound bilateral hearing loss (pure tone detection thresholds at 80 dB HL or greater at octave frequencies ranging from 0.5 to 8 KHz). The principal method of communication for all CI users was oral/lip-reading. Pure-tone detection thresholds were within normal limits (30 dB HL or less) at frequencies ranging from 250 to 6000 Hz for all CI users and control group participants. CI users were separated in two groups according to the length of experience with the implant. In accordance to previous assessments of perceptional performance and duration of implant use (Allen et al., 1998; Damen et al., 2007), duration of CI use for those individuals with less than 6 years of experience was classified as “short-term” (*n* = 11) and those with more than 6 years were classified as having “long-term” experience (*n* = 8). The Research Ethics Board of the Université de Montréal approved the study and all the participants provided written informed consent.

**Table 1:** Clinical profile of cochlear implant users.

Participants	Sex	Age	Age at onset of deafness (years)	Cause of deafness	Deafness duration (years)	Speech recognition (%)	Duration with the implant (years)
S1	F	46	0 (sudden)	Hereditary	43	0	3
S2	F	65	16–62 (progressive)	Hereditary	46	76	3
S3	M	40	7–35 (progressive)	Unknown	30	90	3
S4	F	49	17–38 (progressive)	Hereditary	21	76	3
S5	M	22	0 (sudden)	Unknown	18	80	4
S6	F	35	0 (sudden)	Hereditary	31	82	4
S7	F	32	0–14 (progressive)	Hereditary	14	84	4
S8	F	56	16–50 (progressive)	Hereditary	34	92	5
S9	F	58	0 (sudden)	Hereditary	53	20	5
S10	F	43	14–33 (progressive)	Ototoxic	24	78	5
S11	F	57	0–52 (progressive)	Hereditary	52	72	5
L1	M	58	10–33 (progressive)	Ototoxic	42	84	6
L2	F	44	0 (sudden)	Hereditary	38	56	6
L3	M	48	0–39 (progressive)	Hereditary	41	80	7
L4	M	65	14 (sudden)	Infectious	42	66	8
L5	F	63	7–11 (progressive)	Hereditary	25	54	8
L6	F	38	0 (sudden)	Unknown	30	20	8
L7	F	36	12–26 (progressive)	Unknown	14	78	9
L8	M	24	0 (sudden)	Hereditary	13	2	9

*S = short-term experience (group 1); L = long-term experience (group 2).*

### STIMULI, DESIGN, AND PROCEDURE

Prior to testing, tactile and auditory capabilities were evaluated to further ensure unisensory homogeneity for both groups. A static two-point discrimination evaluation was performed for each participant to ensure normal to fair innervation. Five one-point and five two-point contacts at a set distance were presented in random order on the right index finger. Participants were required to correctly identify the number of points for seven of ten applications. All eligible participants were confirmed to possess normal to fair (two-point distances between 6 and 10 mm) right index finger innervation density (Warwick et al., 2009). Tactile sensitivity thresholds were tested for all participants using Semmes-Weinstein monofilaments (Bell-Krotoski and Tomancik, 1987). All participants were able to detect a pressure of 2.83 g/mm^2^ on their right index fingers and deemed to have normal tactile sensitivity thresholds. Two additional tactile evaluations were conducted. Right index tactile resolution was tested using a grating orientation task in which domes of varying grating widths were presented at random orientations (Van Boven and Johnson, 1994). Participants were asked to assess the dome’s orientation as either parallel or perpendicular using only tactile cues. The grating width at which participants would correctly identify the orientation for 75% of presentations was then calculated. Vibrotactile discrimination thresholds were calculated using a 2-down 1-up staircase method. Participants were presented two consecutive vibrotactile stimuli to their right index fingers and asked if they were identical or different (Alary et al., 2009). Results from the staircase method were used to calculate mean vibrotactile discrimination thresholds. A 3 × 2 ANOVA with group (control; short-term CI users; long-term CI users) as a between-subjects factor and conditions (grating orientation task; vibrotactile discrimination task) as a within-subjects factor was conducted. As expected, there was no main effect for groups [*F*(2,35) = 2.454, *p* = 0.101, $h_{p}^{2}$ = 0.123] and the interaction between factors was not significant [*F*(2,35) = 2.147, *p* = 0.132, $h_{p}^{2}$ = 0.109].

For the main task, participants sat in a comfortable chair in a sound-attenuated booth. They were asked to rub the palms of their hands together back and forth four times at approximately 2 cycles per second in front of a microphone. In accordance with the methods of Jousmäki and Hari (1998), the sounds produced by the rubbing of their hands were played back to them in real time through attenuating circumaural headphones (10 S/DC, David Clark, Worcester, MA, USA) at a self-adjusted comfortable hearing level (between 50 and 60 dB HL) for all participants. For CI users, the headphones were positioned in a normal fashion with the speaker over the CI’s microphone located behind the helix of the pinna. During the experiment, three different auditory conditions were used (for an explicit detailing of the experimental procedure, see Champoux et al., 2011). In the first experimental condition, the auditory stimulus was the unaltered recorded sound. In the second and third conditions, the sounds were modified with an equalizer (Realistic, model 31-2018A) and a mixer (Yamaha, MG10/2 mixing console). In the second condition, the audio feedback was accentuated by 20 dB and the frequencies above 2 kHz were increased by an additional 12 dB. In the third condition, audio feedback was reduced by 20 dB and frequencies above 2 kHz were attenuated by an additional 12 dB. According to Jousmäki and Hari (1998), the second and third conditions induce the perception of drier and moister palmar skin, respectively. The three experimental conditions were each repeated ten times in a pseudorandom order.

Participants were informed to focus on tactile perception and to report any perceived changes relating to palmar skin sensation on a scale of /+5/to/-5/, where /+5/ represented dryness and /-5/ represented moistness. Before the start of the experiment, participants rubbed their palms together with the instruction to remember their sensation as “a normal palmar skin perception” (i.e., number /0/ on our scale). They were specifically instructed to report changes in tactile sensation and not auditory perception. Participants reported their responses verbally to the experimenter. The number /0/ referred to a normal degree of moisture–dryness of the palmar skin, /-5/ suggested that palmar skin felt moister whereas /+5/ suggested that palmar skin felt drier. In their original experiment of the parchment-skin illusion, Jousmäki and Hari (1998) used a similar scale to assess a range of rough/moist to smooth/dry values. However, a multi-dimensional scale such as that used by Jousmäki and Hari (1998) may generate confusion in the response (Guest et al., 2002). Furthermore, the rough–smooth scale has been evaluated independently and has proved to be more difficult to interpret than the dry–moist scale (Guest et al., 2002). As such, the present study made use of a uni-dimensional scale (dry-moist) to minimize any potential ambiguities in qualifying palmar skin changes. As in previous investigation using the exact same procedure (see Champoux et al., 2011), non-parametric statistics were used, as it is designated for datasets without a uniform response criterion and when using scale ratings (in this case, /-5/to/+5/).

## RESULTS

**FIGURE 1:**
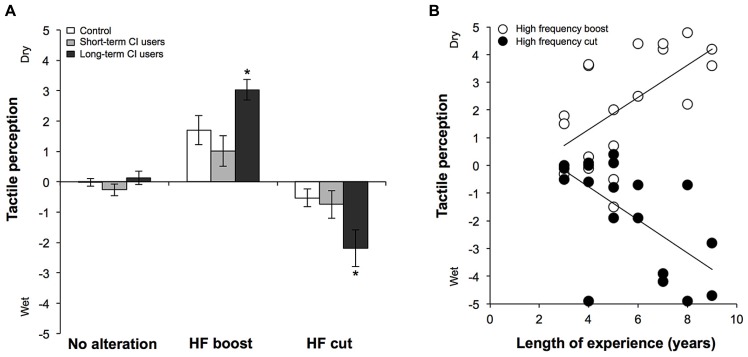
**(A)** Changes in palmar skin perception during the parchment-skin illusory task in the control, short-term and long-term CI users without modification of the auditory signal (no alteration), with accentuated high frequencies (HF boost) or with attenuated high frequencies (HF cut). **(B)** Individual results of CI users in the two experimental conditions (HF boost and HF cut). The data reveals that CI users with less experience with the implant perceive significantly less change in tactile sensation compared to individuals with more experience. * *p* < 0.05.

All participants were able to accurately identify the condition referred to as “a normal palmar skin perception”. In this condition without auditory modification, the reported perception was continuously very close to /0/ and only had small variations in the responses (see **Figure 1A**). The results show that a parchment-skin illusion was clearly perceived by each group. Indeed, all individuals consistently reported a clear change in palmar skin perception whenever the high frequencies were increased or decreased (**Figure 1A**). As expected, palmar skin was reported to be dryer in the second condition and moister in the third. The performance in the “long-term” CI users group, however, appeared greater in theses conditions compared to the performance of the control and the “short-term” CI users group.

We first conducted a Mann-Whitney test in order to reveal any difference between the control group and CI users, without distinction to the duration of CI use, for the experimental conditions. When all CI users were confounded, there was a significant different between tactile sensations when high frequencies were attenuated (*U* = 107.0; *p* = 0.030). No significant differences were found between groups when auditory stimuli were not modified (*U* = 164.5; *p* = 0.638) or when high frequencies were amplified (*U* = 158.0; *p* = 0.511). Then, as in previous research using the same experimental technique (see Champoux et al., 2011), we performed a Kruskal–Wallis ANOVA between groups (control; “long-term CI users; “short-term CI users) for the three experimental conditions (no alteration; high-frequency boost; high-frequency cut). There was a significant difference between the changes in tactile sensation, both when high frequencies were amplified [*c*^2^(2) = 9.52; *p* = 0.009] or attenuated [*c*^2^(2) = 11.67; *p* = 0.003]. As expected, there was no significant difference between groups when the auditory stimuli were not modified [*c*^2^(2) = 2.68; *p* = 0.262]. *Post hoc* Mann–Whitney tests revealed that the perceived changes in palmar skin for the “long-term” CI users groups were significantly different from those of the control and the “short-term” CI users groups. Significant differences were found between “long-term” CI users and control individuals, whether higher frequencies were amplified (*U* = 14.5; *p* = 0.022) or reduced (*U* = 33.0; *p* = 0.001). The same was also observed between “long-term” CI users and “short-term” CI users whether the higher frequencies were amplified (*U* = 5.5; *p* = 0.001) or reduced (*U* = 12.0; *p* = 0.008). There was no significant difference in palmar skin perception between control and “short-term” CI users whether the higher frequencies were amplified (*U* = 84.0; *p* = 0.377) or reduced (*U* = 92.5; *p* = 0.599). After correcting for multiples comparisons (corrected *p*-value = 0.0125), we predictably found a significant relation between the length of experience with the implant and the reported change in the tactile perception (**Figure 1B**) whether higher frequencies were amplified (*r* = 0.585; *p* = 0.009) or reduced (*r* = -0.702; *p* = 0.001). We were unable to find any other significant relationships between performance in audiotactile conditions and the characteristics of hearing loss (i.e., age at the onset of deafness, deafness duration and speech recognition score with the CI).

## DISCUSSION

In the present study, we examined spectral audiotactile interaction capabilities of CI users. Consistent with previous results, these data confirm that a prolonged period of deafness followed by cochlear implantation can lead to abnormal audiotactile interactions (Landry et al., 2013). Moreover, our results suggest that length of CI use might be an important factor related to audiotactile performance. This is consistent with the general assumption that longer periods of experience with a CI might lead to restored sensory functions (e.g., Allen et al., 1998; O’Donoghue et al., 1998; Nicholas and Geers, 2006; Pantev et al., 2006; Damen et al., 2007). The results are also in agreement with data suggesting important tactile-to-auditory changes following deafness and that auditory experience plays an important role in efficient cross-modal processing. Indeed, evidence of altered susceptibility to auditory-tactile illusions suggests two important facets of multisensory interaction in relation to temporary deafness. First, constant auditory input is necessary from birth for the proper development of normal-like audiotactile interactions. Second, auditory and tactile information is seemingly processed differently in CI users.

Despite the apparent similarities between results from both audiotacile interaction studies conducted in our laboratory, some important distinctions must be emphasized. Our previous investigation suggests that CI users are able to easily ignore auditory stimuli in a temporal cross-modal segregation task compared to controls, regardless of the duration of CI use (Landry et al., 2013). Contrarily, the results of the present study suggest that as they become more experienced, CI users are increasingly influenced by auditory stimuli in a spectral cross-modal fusion task. Taken together, these results support the notion that CI users have abnormal overall multisensory interactions. However, these combined data underline why a general statement as to whether CI users are better or worse multisensory integrator will most probably never be entirely valid. It appears that the directionality of the results obtained is dependent on a variety of factors, such as the examined sensory modalities, task directives, CI proficiency, and the characteristics related to hearing loss. Hence, multisensory data for CI users needs to be considered in the context of the specificity of the task along with the modalities examined.

A number of human and non-human primate studies have investigated cortical regions involved in the convergence of auditory and somatosensory processing (e.g., Foxe et al., 2000, 2002; Fu et al., 2003; Murray et al., 2005; Caetano and Jousmäki, 2006; Schürmann et al., 2006; Hackett et al., 2007; Lakatos et al., 2007). These studies suggest an interaction of auditory and tactile inputs in cortical areas such as primary and associative auditory regions which were traditionally assumed to be unimodal. After auditory deprivation, the brain can reorganize so that the deprived sensory cortex increasingly processes tactile stimuli. Indeed, imaging data suggests that vibrotactile stimuli can activate auditory regions in the deaf (Levänen et al., 1998; Schürmann et al., 2006; Sharma et al., 2007) and cortical over-representation of somatosensory evoked potentials in the left temporal region was found in deaf children using a CI (Charroó-Ruíz et al., 2013). Several data demonstrate that brain reorganization induced by deafness leads to behavioral changes for numerous perceptual tasks (Hanson, 1982; Neville and Lawson, 1987; Loke and Song, 1991; Bavelier et al., 2000, 2001, 2006; Bosworth and Dobkins, 2002; Heming and Brown, 2005; Turgeon et al., 2012), although it is unsure whether behaviorally advantageous (e.g., Bolognini et al., 2012). The effect of cross-modal reorganization raises important questions on the importance of hearing experience in shaping perceptual processing, but also in regards to cochlear implantation. It is now generally accepted that brain reorganization is likely a factor restricting access to auditory stimulation in long-term deafened individuals following cochlear implantation (e.g., Naito et al., 1997; Giraud et al., 2001; Lee et al., 2001; Green et al., 2005; Doucet et al., 2006). In light of the possibility that visual and tactile input may be redirected to auditory cortical areas, the question of how these modalities interact during tasks that require multisensory processing following cochlear implantation is of great interest.

Research on multisensory interaction has suggested ease for CI users when using congruent cues (Tyler et al., 1997; Giraud et al., 2001; Kaiser et al., 2003; Geers, 2004; Bergeson et al., 2005; Moody-Antonio et al., 2005). Some researchers have even gone as far as to suggest that CI users could be better than hearing individuals at integrating audiovisual information (e.g., Rouger et al., 2007). However, given the apparent invasion of the auditory cortex by visual or tactile information, it could be hypothesized that visual or tactile information might interferes with auditory treatment when stimuli from these modalities are incongruent. The ability to fuse incongruent audiovisual information has been studied by Schorr et al. (2005). They used McGurk-like stimuli (see McGurk and MacDonald, 1976) to investigate the ability to integrate incongruent multisensory cues in children with CI as a function of experience with spoken language. The authors found normal-like results for the audiovisual task in children aged two and a half years or younger. Conversely, the fusion capability in children implanted later in life was reduced. This is consistent with the notion that duration of deafness influences cortical reorganization and has an impact on CI proficiency. The ability of CI users to fuse and segregate conflicting auditory and visual information has been investigated with speech and non-speech tasks (e.g., Champoux et al., 2009; Tremblay et al., 2010; Landry et al., 2012). It is essential to consider this potential difficulty for CI users to interpret audiovisual information in conjunction with investigations of other cross-modal interactions, such as audiotactile, to form a complete view of multisensory interactions in CI users. The data from the examination of audiotactile cross-modal segregation capabilities in CI users (Landry et al., 2013) and the one conducted in the present studies using a cross-modal fusion task are in complete agreement with the outcomes from studies in the audiovisual domain. Indeed, these data suggest that while non-auditory signals can facilitate auditory perception in some multisensory conditions (i.e., in cross-modal fusion tasks), they may hinder discrimination performance for some CI users when multisensory inputs require segregation. The aforementioned investigations highlight the potential changes to tactile-to-auditory interactions following profound deafness. These observed change in cross-modal performance require interpretation in relation to factors related to deafness as factors of hearing loss seem to play a considerable role in the extensive cross-modal changes.

Several deafness and implantation factors have been shown to influence CI performance (see Collignon et al., 2011). Our data suggest that of these factors, spectral audiotactile interaction might be influenced more significantly by duration of CI use. This lends credence to the notion that a greater span of experience with the implant might help re-establish sensory functions after a prolonged deprivation (e.g., Nicholas and Geers, 2006; Pantev et al., 2006). However, we found no relationship between any other of the characteristics of the hearing loss and the examined multisensory performance. Thus, the data suggest that neither age at the onset of deafness, the duration of auditory deprivation, or CI proficiency had an impact on spectral audiotactile interaction. However, the composition of the group regarding the many characteristics of the hearing loss and CI use may explain why no significant differences were found for these factors. First, all participant had more than a decade of auditory deprivation and were implanted at least at 15 years of age. Second, although some participants were congenitally deaf, all participants continuously used hearing devices before cochlear implantation, possibly preserving a minimal degree of auditory inputs during this period. Finally, CI speech perception proficiency was almost identical between groups, with the exception of two participants. These limitations could explain why no significant relationship was found between the results and performance with the CI or characteristics of hearing loss.

Cochlear implant user results for the parchment-skin illusion are constant with the notion that continuous auditory input from birth seems to be necessary for the maintenance of normal auditory interactions. The results presented in this study contribute to the burgeoning literature regarding the effects of a temporary auditory deprivation on the emergence, development, and maintenance of normal-like multisensory processes. However, further experiments comprising groups of deaf individuals with more homogeneous characteristic of hearing loss and CI use will be needed in order to support the implication of each feature of hearing loss in multisensory processing. The functional implications for the alterations observed in this study also merit further investigations; as such abnormal interactions could prove to be either beneficial or detrimental depending of perceptual situations.

## Conflict of Interest Statement

The authors declare that the research was conducted in the absence of any commercial or financial relationships that could be construed as a potential conflict of interest.
